# The Association Between Serum/Plasma Leptin Levels and Obstructive Sleep Apnea Syndrome: A Meta-Analysis and Meta-Regression

**DOI:** 10.3389/fendo.2021.696418

**Published:** 2021-09-24

**Authors:** Xiaoyan Li, Jie He

**Affiliations:** ^1^ Department of endocrinology, Clinical Medical College and The First Affiliated Hospital of Chengdu Medical College, Chengdu, China; ^2^ Department of Pulmonary and Critical Care Medicine, Clinical Medical College and The First Affiliated Hospital of Chengdu Medical College, Chengdu, China

**Keywords:** leptin, obstructive sleep apnea syndrome, meta-analysis, obesity, serum, plasma

## Abstract

**Background:**

Obstructive sleep apnea syndrome (OSAS) is associated with various adipokines. Leptin, a common adipokine, has attracted considerable attention of many researchers in recent years. So far, there has been little agreement on whether blood leptin levels differ in patients with OSAS. Thus, this meta-analysis examined the relationship between serum/plasma leptin levels and the occurrence of OSAS.

**Method:**

WanFang, Embase, CNKI, Medline, SinoMed, Web of Science, and PubMed were searched for articles before March 30, 2021, with no language limitations. STATA version 11.0 and R software version 3.6.1 were used to analyze the obtained data. The weighted mean difference and correlation coefficients were used as the main effect sizes with a random-effects model and a fixed-effects model, respectively. Trial sequential analysis was conducted using dedicated software.

**Result:**

Screening of 34 publications identified 45 studies that met the inclusion criteria of this meta-analysis and meta-regression. Our results suggested that plasma/serum leptin levels were remarkably higher in individuals with OSAS than in healthy individuals. Subgroup analyses were performed based on OSAS severity, ethnicity, age, body mass index, assay type, and sample source. The serum and plasma leptin levels were increased in nearly all OSAS subgroups compared to those in the corresponding control groups. Meta-regression analysis indicated that age, BMI, severity, assay approaches, study design, PSG type and ethnicity did not have independent effect on leptin levels. Furthermore, a positive relationship between the serum/plasma leptin level and apnea-hypopnea index (AHI) was found in the meta-analysis. The results of the trial sequential analysis suggested that the enrolled studies surpassed the required information size, confirming that our study findings were reliable.

**Conclusion:**

Our study results demonstrate that OSAS patients have higher leptin levels in serum/plasma compared to controls, and the serum/plasma leptin level is positively correlated with AHI, especially in adults.

## 1 Introduction

Obstructive sleep apnea syndrome (OSAS) is a multifactorial disease with complex pathophysiology that manifests as upper airway obstruction, chronic nocturnal intermittent hypoxia, and fragmented sleep (1). Numerous studies have shown that OSAS is a primary independent predictor of cardiovascular disease and is strongly linked to metabolic syndrome, insulin resistance, and obesity (2, 3). The incidence of moderate-to-severe OSAS has been estimated to be up to 23.4% in women and 49.7% in men, with the increasing prevalence over the past two decades (4). In addition, the OSAS prevalence is 12- to 30-fold higher in the obese population than in the non-obese population (5). As much as 60-70% of patients with OSAS have comorbid obesity, particularly visceral obesity (6).

Leptin is a common adipocytokine that is produced and secreted by white adipose tissue (7). Leptin’s primary effect is signaling satiety and decreasing the motivation to consume food. In addition, leptin and the leptin receptor are involved in energy expenditure, balance of blood glucose metabolism, inflammatory processes, and regulation of immune function (8). Levels of circulating leptin are significantly increased in the obese state of the body. Obesity is probably the most critical risk factor for OSAS progression, which is common among adolescents (9). Numerous studies have indicated the development or worsening of OSAS with increasing weight, as opposed to extensive improvement with weight loss (10). Kapusuz et al. (11) showed that patients with OSAS have higher circulating levels of leptin compared to controls. But the association between OSAS and serum/plasma leptin levels is intricate and multidirectional because obesity alone can also affect leptin levels. Sánchez et al. (12) reported that obese individuals without OSAS also display higher circulating levels of leptin, and sleep apnea is not a decisive factor for leptin levels. Similarly, Ursavas et al. (13) observed no differences in serum leptin between OSAS patients and controls. Moreover, some studies that directly examined the relationship between OSAS and leptin serum/plasma levels have been recently published, yet with controversial conclusions (14, 15).

Therefore, it is necessary to conduct a meta-analysis using all available relevant and new studies to quantitatively evaluate the relationship between serum/plasma leptin levels and the occurrence of OSAS. To the best of our knowledge, we enrolled in this study all publications that covered most comprehensive samples. Moreover, this is the first systematic review and meta-analysis to determine the pooled correlation coefficient of OSAS and apnea-hypopnea index (AHI).

## 2 Materials and Methods

### 2.1 Identification of Eligible Studies and Data Screening

We conducted a literature search for articles that measured leptin in serum/plasma samples from individuals with OSA and controls. And our meta-analysis was registered in the PROSPERO system (ID: CRD42021245786). The Web of Science, WanFang, Embase, SinoMed, Medline, PubMed, and CNKI databases were screened to identify relevant past studies (up to March 30, 2021). The keywords and subject terms employed in the search included: “leptin” or “LEP” combined with “obstructive sleep apnea” or “OSA” or “OSAS”. All references in these articles were inspected to identify additional articles that were not retrieved by the initial electronic database search. Eligible studies were required to meet the following inclusion criteria: (1) a case-control, cohort, or cross-sectional study; (2) leptin serum/plasma levels measured in individuals with OSAS without restrictions on sex, nationality, age, or ethnicity; and (3) subjects met the OSAS diagnostic criteria based on polysomnography (adults: an AHI of ≥5/h; children: an AHI of >1/h). The exclusion criteria were as follows: (1) commentaries, letters, editorials, other types of literature reviews, or case reports, and (2) adequate information could not be extracted from the original articles, and the study authors could not be contacted to provide more detailed data.

### 2.2 Study Selection

According to the abovementioned retrieval strategy, two authors searched the above databases independently and read the titles and abstracts of identified articles. We obtained full-text articles of all potentially eligible abstracts. Then, we re-evaluated the prospectively appropriate articles by abstracting and evaluating the full text in detail. If no consensus was reached concerning the inclusion or exclusion of an article, a third experienced reviewer was consulted.

### 2.3 Data Extraction and Management

We employed a specifically designed table to extract data from each of the enrolled studies as follows: (1) basic data such as first author, publication time; (2) baseline features of the study participants such age, BMI, sample size, ethnicity and sex, serum/plasma leptin levels in patients and controls; (3) disease severity; (4) quality of the literature; and (5) Spearman’s rank correlation coefficient or Pearson product-moment correlation coefficient between AHI and leptin levels.

### 2.4 Methodological Evaluation of the Study Quality

The quality of the included studies was examined using the Newcastle-Ottawa Scale (NOS), which analyses publications regarding the study population (4 items, full score 4), exposure or outcome (3 items, overall score 3), and comparability (1 item, overall score 2). Total scores of 7-9, 4-6, and 0-3 were considered high-, medium-, and low-quality studies, respectively.

### 2.5 Trial Sequential Analysis

Trial sequential analysis (TSA) was performed to assess whether quantitative findings were robust and to calculate the required information size (RIS). As cumulative meta-analyses carry the risk of producing random errors due to sparse data and repetitive testing, we added TSA to the statistical methods. When the cumulative Z-curve crossed the monitoring boundary, the result was regarded as convincing. We used a two-sided trial sequential monitoring boundary and calculated the RIS based on α=0.05 and β=0.20. The mean differences and effect sizes were calculated from the included studies. The software TSA version 0.9.5.10 beta was used for this analysis (Copenhagen Trial Unit, Centre for Clinical Intervention Research, www.ctu.dk/tsa).

### 2.6 Statistical Analyses

In this meta-analysis, we explored the summarized findings of the studies using the R software version 3.6.1 (R Project for Statistical Computing, https://www.r-project.org/) and STATA version 11.0 (Stata Corporation, College Station, TX, USA). Continuous outcomes were harmonized and expressed as weighted mean differences (WMDs) with 95% confidence intervals (95%CIs). Given that the standard error mostly depends on the significance of the rank correlation coefficient, the dependence on the sampling distribution of the Pearson product-moment correlation coefficient is not suggested. We compared each correlation coefficient using the Fisher transformation and then conducted an analysis with the transformed values as the input before their conversion back to correlation coefficients (CORs). Cohen’s criteria were applied to assess the computed effect size (small: ≤0.3, moderate: 0.3-0.5, large: >0.5) (16). Associations between AHI and leptin levels were analyzed using Pearson’s correlation coefficient. Based on the previous description, the reported Spearman’s correlation coefficients in some studies were converted into Pearson’s correlation coefficients (17). Cochran’s Q test with chi-square tests and I^2^ were employed to check for data heterogeneity, with heterogeneity cutoff points defined as 75% (high), 50% (moderate), and 25% (low). For nonsignificant heterogeneity, defined as *P*>0.1 and I^2^<50%, a fixed-effects model was chosen; if *P*<0.1 along with I^2^>50% indicated heterogeneity among the data, a random-effects model was employed. Subgroup analysis, descriptive analysis, or meta-regression was often used for heterogeneity analysis. Subgroup analyses were performed for ethnicity, study design, study quality, and type of disease. A sensitivity test was conducted to explore the influence of each study on the pooled WMD by eliminating one study at a time. Moreover, publication bias was examined using Begg’s and Egger’s tests as quantitative methods.

## 3 Results

### 3.1 Literature Search and Enrolled Studies

**Figure 1:**
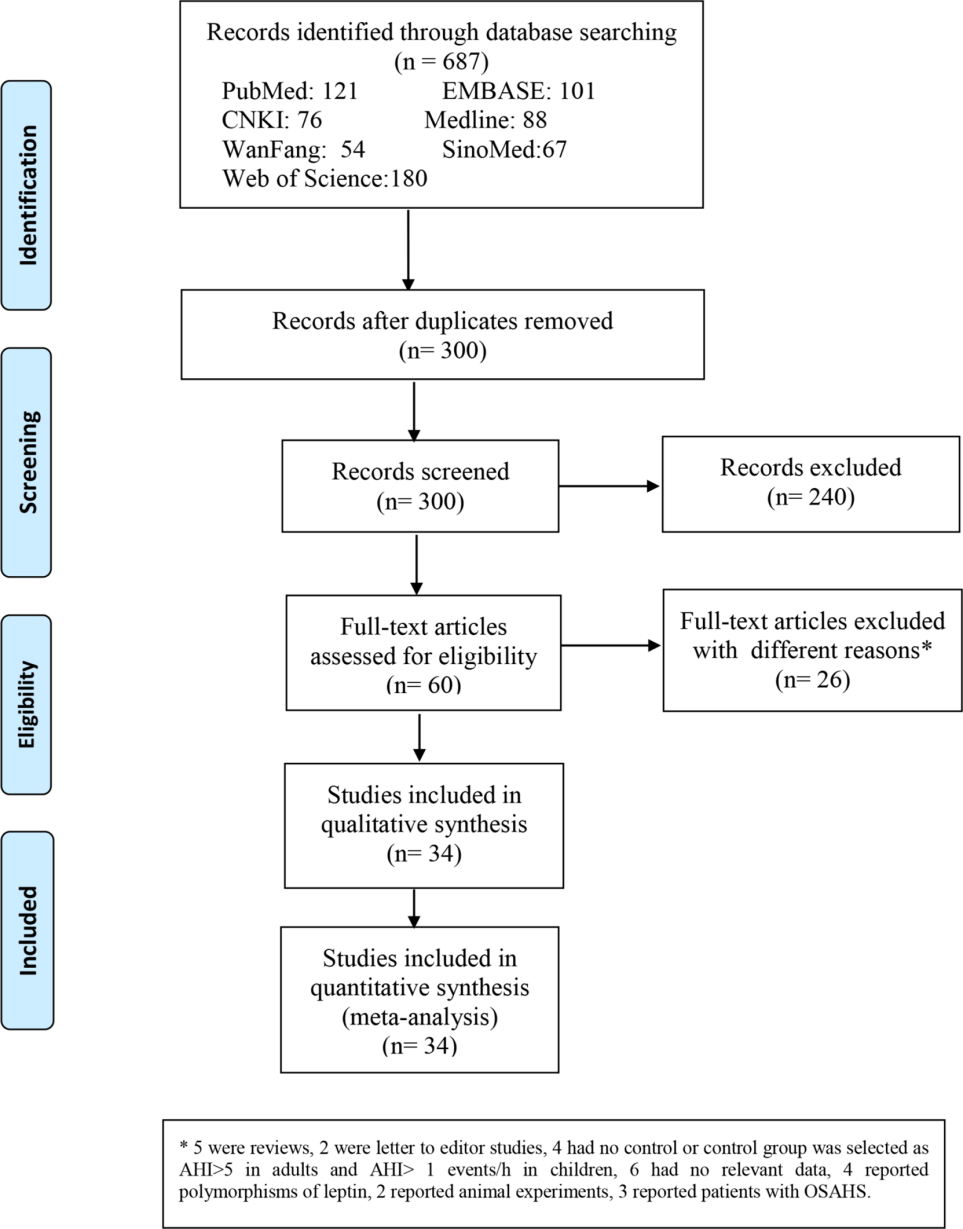
Flow diagram indicating the literature selection process and results based on the preferred reporting items for meta-analyses.

Overall, 687 relevant articles were extracted from the databases. After the screening of titles and abstracts and the omission of duplicates, 627 articles were excluded, and 60 publications were included in the study. We retained 60 articles in which full texts were retrieved from the databases. The full-text articles were reviewed meticulously based on the inclusion and exclusion criteria described in the Methods section. Another 26 articles were excluded for the reasons indicated in **Figure 1**. We retrieved the full texts of the articles and excluded several full texts for following reasons: five were reviews; two were letter to editor studies; four had no control or control group was selected as AHI>5 events/h in adults and AHI>1 events/h in children; six had no relevant data; four reported polymorphisms of leptin; 2 reported animal experiments;3 reported patients with OSAS. Then another 26 articles were excluded for the different reasons indicated in **Figure 1**. As shown in **Table 1**, three were conducted in China (18, 29, 45), five in the USA (14, 34, 42), four in Poland (19, 21, 31), nine in Turkey (11, 13, 20, 32, 35, 38, 44, 46), five in Spain (12, 22, 37), one in Australia (23), three in India (24, 40), three in Greece (26, 30), three in Italy (33, 41), one in Brazil (36), two in Belgium (43), one in Egypt (15). With regard to ethnicity, 33 studies were performed with Caucasians (11–14, 19–23, 26, 30–35, 37, 38, 41–44, 46), 11 with Asians (18–25, 29, 39, 40, 45), and one with Latinos (36) of the 45 studies, 13 reported leptin levels in plasma (12, 14, 19, 22, 30, 33, 42, 46), and 32 (11, 13, 15, 20, 21, 23–29, 31, 32, 34–41, 43–45) reported leptin levels in serum. One is cohort study (24). Thirteen are cross-sectional study (13, 26, 28, 32, 37, 39, 40, 42, 43). Thirty-one are case-control study (11, 12, 14, 15, 18–23, 25, 27, 29–31, 33–36, 38, 41, 44–46). Nine studies included children (14, 29, 34, 42, 43, 45). The data of leptin levels, age, BMI, severity degree and AHI are summarized in **Table 2**. Eight studies provided Pearson’s or Spearman’s correlation coefficient between leptin and AHI (12, 18, 20, 25, 27, 29, 38, 46) (**Table 2**). The process of the published literature selection is shown in a PRISMA flow diagram (**Figure 1**).

**Table 1:** Characteristics of included studies.

Author	Year	Country	Ethnicity	Case/Control (n)	Leptin Source	Assay approach	NOS	Study design	PSG type
Ip MS et al. (18)	2000	China	Asian	30/30	Serum	RIA	7	CCS	Lab PSG
Phillips BG et al. (19)	2000	Poland	Caucasian	32/32	Plasma	RIA	7	CCS	Lab PSG
Ulukavak Ciftci T et al. (20)	2005	Turkey	Caucasian	30/22	Serum	ELISA	8	CCS	Lab PSG
Rubinsztajn R et al. (21)	2005	Poland	Caucasian	36/15	Serum	RIA	6	CCS	Lab PSG
Barcelo A et al.(obese) (22)	2005	Spain	Caucasian	23/19	Plasma	RIA	8	CCS	Lab PSG
Barcelo A et al.(non-obese) (22)	2005	Spain	Caucasian	24/18	Plasma	RIA	8	CCS	Lab PSG
McArdle N et al. (23)	2007	Australia	Caucasian	21/21	Serum	ELISA	7	CCS	Portable device
Sharma SK et al. (24)	2007	India	Asian	40/40	Serum	ELISA	8	CS	Lab PSG
Tokuda F et al.(mild-to-moderate) (25)	2008	Japan	Asian	21/15	Serum	ELISA	8	CCS	Portable device
Tokuda F et al.(severity) (25)	2008	Japan	Asian	32/15	Serum	ELISA	8	CCS	Portable device
Kapsimalis F et al.(mild-to-moderate) (26)	2008	Greece	Caucasian	26/15	Serum	RIA	7	CSS	Lab PSG
Kapsimalis F et al.(severity) (26)	2008	Greece	Caucasian	26/15	Serum	RIA	7	CSS	Lab PSG
Makinodan K et al. (27)	2008	Japan	Asian	13/12	Serum	ELISA	7	CCS	Lab PSG
Yamamoto Y et al. (28)	2008	Japan	Asian	10/31	Serum	ELISA	6	CSS	Lab PSG
Jian Y et al. (29)	2008	China	Asian	68/30	Serum	RIA	6	CCS	Lab PSG
Antonopoulou S et al. (30)	2008	Greece	Caucasian	45/25	Plasma	ELISA	7	CCS	Lab PSG
Wysocka E et al.(obese) et al. (31)	2009	Poland	Caucasian	18/18	Serum	ELISA	8	CCS	Lab PSG
Wysocka E et al.(non-obese) (31)	2009	Poland	Caucasian	18/18	Serum	ELISA	8	CCS	Lab PSG
Acioglu E et al. (32)	2010	Turkey	Caucasian	34/19	Serum	ELISA	7	CSS	Lab PSG
Ursavas A et al. (13)	2010	Turkey	Caucasian	45/15	Serum	ELISA	7	CSS	Lab PSG
Carpagnano GE et al.(obese) (33)	2010	Italy	Caucasian	36/24	Plasma	ELISA	7	CCS	Lab PSG
Carpagnano GE et al.(non-obese) (33)	2010	Italy	Caucasian	28/20	Plasma	ELISA	7	CCS	Lab PSG
Canapari CA et al. (34)	2011	USA	Caucasian	15/16	Serum	RIA	8	CCS	Lab PSG
Basoglu OK et al. (35)	2011	Turkey	Caucasian	36/34	Serum	ELISA	8	CCS	Lab PSG
Sanchez-de-la-Torre M et al.(obese) (12)	2012	Spain	Caucasian	10/28	Plasma	RIA	7	CCS	Portable device
Sanchez-de-la-Torre M et al.((non-obese) (12)	2012	Spain	Caucasian	21/20	Plasma	RIA	7	CCS	Portable device
Matos G et al. (36)	2013	Brazil	Latinos	119/187	Serum	ELISA	7	CCS	Lab PSG
Salord N et al. (37)	2014	Spain	Caucasian	26/13	Serum	ELISA	8	CSS	Lab PSG
Yosunkaya S et al. (38)	2015	Turkey	Caucasian	31/25	Serum	ELISA	7	CCS	Lab PSG
Chihara Y et al. (39)	2015	Japan	Asian	39/15	Serum	RIA	8	CSS	Lab PSG
Dubey A et al.(mild) (40)	2015	India	Asian	26/22	Serum	ELISA	7	CSS	Lab PSG
Dubey A et al.(moderate-to-severity) (40)	2015	India	Asian	64/22	Serum	ELISA	7	CSS	Lab PSG
De Santis S et al. (41)	2015	Italy	Caucasian	26/24	Serum	ELISA	7	CCS	Lab PSG
Gaines J et al.(mild) (42)	2016	USA	Caucasian	109/138	Plasma	ELISA	8	CSS	Lab PSG
Gaines J et al.(moderate) (42)	2016	USA	Caucasian	44/138	Plasma	ELISA	8	CSS	Lab PSG
Kapusuz Gencer Z et al. (11)	2017	Turkey	Caucasian	36/22	Serum	ELISA	8	CCS	Lab PSG
Van Eyck A et al.(mild) (43)	2017	Belgium	Caucasian	28/111	Serum	ELISA	8	CSS	Lab PSG
Van Eyck A et al.(moderate-to-severe) (43)	2017	Belgium	Caucasian	25/111	Serum	ELISA	8	CSS	Lab PSG
Cakir I et al.(mild-to-moderate) (44)	2018	Turkey	Caucasian	11/30	Serum	ELISA	7	CCS	Lab PSG
Cakir I et al.(severity) (44)	2018	Turkey	Caucasian	39/30	Serum	ELISA	7	CCS	Lab PSG
Ji L et al. (45)	2019	China	Asian	24/24	Serum	ELISA	6	CCS	Lab PSG
Sertogullarindan B et al. (46)	2019	Turkey	Caucasian	55/35	Plasma	ELISA	8	CCS	Lab PSG
Dalesio NM et al.(non-obese) (14)	2020	USA	Caucasian	9/18	Plasma	Mass spectrometry	8	CCS	Lab PSG
Dalesio NM et al.(obese) (14)	2020	USA	Caucasian	13/18	Plasma	Mass spectrometry	8	CCS	Lab PSG
Emara TA et al. (15)	2021	Egypt	Caucasian	23/18	Serum	ELISA	7	CCS	Lab PSG

CSS, cross sectional studies; CCS, case-control studies; CS, cohort studies; PSG, polysomnogram; NOS, Newcastle-Ottawa scale; ELISA, Enzyme linked immunosorbent assay; RIA, Radioimmunoassay; NA, not available.

**Table 2:** Participants’ characteristics of included studies.

Author	Leptin (ng/ml) (Mean **±** SD)		BMI(kg/m^2^) (Mean **±** SD)		Age (years) (Mean **±** SD)		AHI (Mean **±** SD)		R*
	Case	Control	Case	Control	Case	Control	Case	Control	
Ip MS et al. (18)	9.18 ± 4.24	6.54 ± 3.81	27 ± 2.9	26.5 ± 2.1	43.6 ± 10.1	41.9 ± 7.4	35.7 ± 18	1.8 ± 1.9	0.39
Phillips BG et al. (19)	13.7 ± 7.35	9.2 ± 6.79	33 ± 1	32 ± 1	43 ± 2	38 ± 2	47 ± 7	6 ± 1	
Ulukavak Ciftci T et al. (20)	29.42 ± 13.51	20.02 ± 13.45	32.12 ± 4.05	31.03 ± 3.18	NA	NA	44.24 ± 21.99	1.55 ± 0.96	0.38
Rubinsztajn R et al. (21)	15.55 ± 11.26	10.34 ± 6.86	NA	NA	51.47 ± 8.95	44.19 ± 14.6	NA	NA	
Barcelo A et al.(obese) (22)	25.4 ± 1.7	24.7 ± 3.5	34.9 ± 0.7	33.6 ± 0.6	47 ± 2	44 ± 3	49 ± 4	2 ± 1	
Barcelo A et al.(non-obese) (22)	11.5 ± 1.6	5.5 ± 0.5	25.9 ± 0.4	25.5 ± 0.5	50 ± 2	47 ± 1	43 ± 2	2 ± 1	
McArdle N et al. (23)	8.17 ± 2.78	4.13 ± 1.35	28.4 ± 3.4	27.9 ± 3.6	46 ± 10.2	46 ± 9.7	40 ± 27	2.8 ± 1.5	
Sharma SK et al. (24)	7 ± 1.97	6.32 ± 2.15	29.8 ± 3.3	29.1 ± 2.3	42.3 ± 8.3	43.3 ± 7.8	32.32 ± 9.21	1.31 ± 0.56	
Tokuda F et al.(mild-to-moderate) (25)	4.85 ± 3.4	4.24 ± 1.78	31.2 ± 4.4	25.11 ± 2.35	46.8 ± 12.0	45.2 ± 15.8	25.9 ± 7.7	3.12 ± 3.09	0.552
Tokuda F et al.(severity) (25)	9.05 ± 5.91	4.24 ± 1.78	26.0 ± 3.5	25.11 ± 2.35	49.1 ± 13.0	45.2 ± 15.8	65.0 ± 17.3	3.12 ± 3.09	
Kapsimalis F et al.(mild-to-moderate) (26)	23.1 ± 21.8	9.4 ± 6.4	29.4 ± 3.8	28.7 ± 4.3	50.5 ± 13.8	47.0 ± 12.5	16.2 ± 5.2	3.1 ± 1.1	
Kapsimalis F et al.(severity) (26)	20.2 ± 17.5	9.4 ± 6.4	30.6 ± 3.4	28.7 ± 4.3	55.3 ± 11.6	47.0 ± 12.5	48.1 ± 15.6	3.1 ± 1.1	
Makinodan K et al. (27)	8.6 ± 3.1	5.9 ± 2.3	28.3 ± 2	26.8 ± 3	43.7 ± 6	40.2 ± 3.6	54.3 ± 20.3	5.1 ± 2.5	0.354
Yamamoto Y et al. (28)	5.52 ± 1.11	5.09 ± 1.31	28 ± 1	27 ± 2	49 ± 2	46 ± 6	33.1 ± 4	3.4 ± 0.4	
Jian Y et al. (29)	12.1 ± 4.9	4.5 ± 0.7	NA	NA	NA	NA	NA	NA	0.451
Antonopoulou S et al. (30)	24 ± 6	8 ± 3	33.5 ± 7	31 ± 3	52 ± 12	51 ± 7	39 ± 25	NA	
Wysocka E et al.(obese) et al. (31)	22.67 ± 13.42	22.86 ± 9.01	33.6 ± 2.8	34.3 ± 2.4	50 ± 8	51 ± 8	36 ± 23.5	1.8 ± 1.5	
Wysocka E et al.(non-obese) (31)	21.21 ± 14.95	21.74 ± 15	28.1 ± 2	28.3 ± 1.7	52 ± 10	50 ± 11	31 ± 20	2.4 ± 0.8	
Acioglu E et al. (32)	44.23 ± 36.38	42.79 ± 25.99	36.28 ± 5.32	35.93 ± 3.93	48.53 ± 7.76	50.42 ± 7.66	45.25 ± 21.94	4.17 ± 1.93	
Ursavas A et al. (13)	10.9 ± 2.7	9.4 ± 0.9	32.5 ± 0.9	31.6 ± 1.8	51.1 ± 1.2	48.4 ± 3	43.5 ± 3.6	2.8 ± 0.4	
Carpagnano GE et al.(obese) (33)	37.91 ± 9.91	44.03 ± 15.3	42.24 ± 0.96	41.6 ± 1.07	36.67 ± 4.02	44.32 ± 2.31	57.56 ± 2.53	4.18 ± 0.33	
Carpagnano GE et al.(non-obese) (33)	30.62 ± 9.69	8.16 ± 3.47	23.96 ± 0.22	23.8 ± 0.42	41.07 ± 3.47	40 ± 2.67	40.55 ± 5.16	3.58 ± 0.24	
Canapari CA et al. (34)	32.19 ± 8.27	21.18 ± 7.77	43.9 ± 13.9	35.4 ± 5.8	12.7 ± 2.64	12.6 ± 2.73	6.26 ± 6.77	0.48 ± 0.3	
Basoglu OK et al. (35)	13.1 ± 1.7	9.01 ± 1.9	33.5 ± 5.7	34.5 ± 2.9	50 ± 19.7	49.7 ± 11.1	27.7 ± 19.6	NA	
Sanchez-de-la-Torre M et al.(obese) (12)	19.27 ± 11.35	16.06 ± 7.22	34.34 ± 3.49	32.01 ± 1.61	46.61 ± 11.03	48.7 ± 9.28	48.92 ± 17.52	2.87 ± 1.51	0.39
Sanchez-de-la-Torre M et al.((non-obese) (12)	7.02 ± 3.24	7.85 ± 3.79	25.02 ± 1.22	24.71 ± 2.39	49.33 ± 10.71	42.9 ± 9.16	41.45 ± 18.3	3.06 ± 1.52	
Matos G et al. (36)	8.32 ± 1.24	5.9 ± 1.37	24.5	29.7	NA	NA	40.7	51.2	
Salord N et al. (37)	47.75 ± 11.39	45.29 ± 6.33	45.33 ± 2.01	44.25 ± 1.79	45.0 ± 3.02	38.81 ± 4.48	47.58 ± 8.58	11.5 ± 1.49	
Yosunkaya S et al. (38)	50.5 ± 17.5	56.3 ± 25.5	32.9 ± 3.6	31.9 ± 2.5	43.3 ± 8.5	41.5 ± 9.5	47.4 ± 19.8	3.4 ± 1.2	0.451
Chihara Y et al. (39)	7.1 ± 4.5	6.5 ± 3.8	26.5 ± 3.9	26.2 ± 3	54.6 ± 12.4	54.3 ± 14.3	26.93 ± 8.06	8.81 ± 1.29	
Dubey A et al.(mild) (40)	9.5 ± 6.9	6.1 ± 3.8	29.3 ± 2.8	28 ± 2.3	42.6 ± 9.7	39.7 ± 10.4	8.4 ± 2.3	1.5 ± 0.9	
Dubey A et al.(moderate-to-severity) (40)	10.3 ± 7.8	6.1 ± 3.8	31.5 ± 3.8	28 ± 2.3	46.7 ± 9.3	39.7 ± 10.4	54 ± 24.2	1.5 ± 0.9	
De Santis S et al. (41)	30.5 ± 10.8	9.25 ± 6.7	33 ± 5.2	30.8 ± 4.3	41.8 ± 7.4	43.7 ± 8.2	26.15 ± 12.1	1.65 ± 0.9	
Gaines J et al.(mild) (42)	12.07 ± 1.22	12.25 ± 1.09	NA	NA	18.41 ± 0.33	16.37 ± 0.18	12.08 ± 0.7	0.79 ± 0.4	
Gaines J et al.(moderate) (42)	16.18 ± 1.9	12.25 ± 1.09	NA	NA	17.44 ± 0.21	16.37 ± 0.18	3.14 ± 0.45	0.79 ± 0.4	
Kapusuz Gencer Z et al. (11)	32.06 ± 4.14	17.77 ± 6.07	34.4 ± 6.9	33.34 ± 6.5	35.1	36.5	37.2 ± 28.8	2.76 ± 1.44	
Van Eyck A et al.(mild) (43)	47.55 ± 19.8	50.81 ± 29.32	NA	NA	10.87 ± 2.73	11.9 ± 2.16	NA	NA	
Van Eyck A et al.(moderate-to-severe) (43)	51.86 ± 21.83	50.81 ± 29.32	NA	NA	12.6 ± 2.8	11.9 ± 2.16	NA	NA	
Cakir I et al.(mild-to-moderate) (44)	2.08 ± 0.49	1.29 ± 0.3	NA	NA	NA	NA	NA	NA	
Cakir I et al.(severity) (44)	2.23 ± 0.9	1.29 ± 0.3	NA	NA	NA	NA	NA	NA	
Ji L et al. (45)	4.27 ± 1.75	2.48 ± 1.61	NA	NA	3.5 ± 1.8	3.6 ± 2.1	NA	NA	
Sertogullarindan B et al. (46)	0.27 ± 0.05	0.20 ± 0.01	32.4 ± 6.7	29.5 ± 5.5	44.44 ± 1.53	42.14 ± 1.89	14.12 ± 4.79	1.94 ± 0.59	-0.231
Dalesio NM et al.(non-obese) (14)	4.5 ± 2.1	7.4 ± 8.8	NA	NA	6 ± 1.8	8.3 ± 2.1	NA	NA	
Dalesio NM et al.(obese) (14)	28.8 ± 21.3	7.4 ± 8.8	NA	NA	7 ± 2.5	8.3 ± 2.1	NA	NA	
Emara TA et al. (15)	18.46 ± 4.73	7.07 ± 1.26	<30	<30	NA	NA	40.83 ± 9.6	NA	

AHI, apnea-hypopnea index; BMI, body mass index; NA, not available; SD, standard deviation; *Pearson’s or Spearman’s correlation coefficient.

### 3.2 Quality Assessment

NOS scores were determined to assess the methodological quality of the included studies. According to these scores, the quality of the eligible literature was relatively high; 30 articles were high-quality studies, whereas the remaining 4 studies were of medium quality (**Table 1**).

### 3.3 Meta-Analysis

#### 3.3.1 Leptin Levels in All Patients With OSA

**Figure 2:**
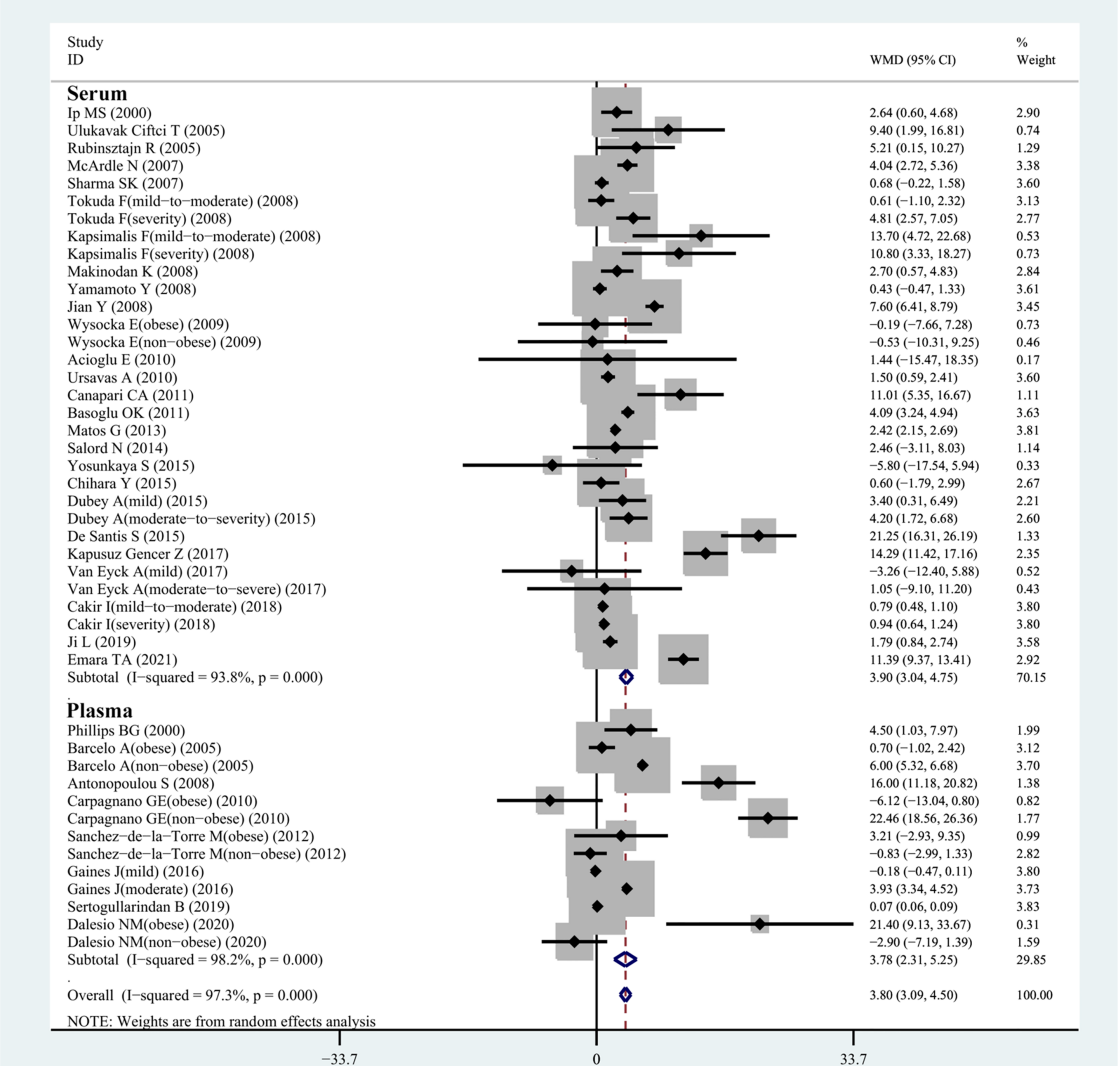
WMD forest plot and 95%CIs for serum leptin levels in the OSAS group in control with the control group in the meta-analysis. 95%CI, 95% confidence interval; OSAS, obstructive sleep apnea syndrome; WMD, weighted mean difference.

**Figure 3:**
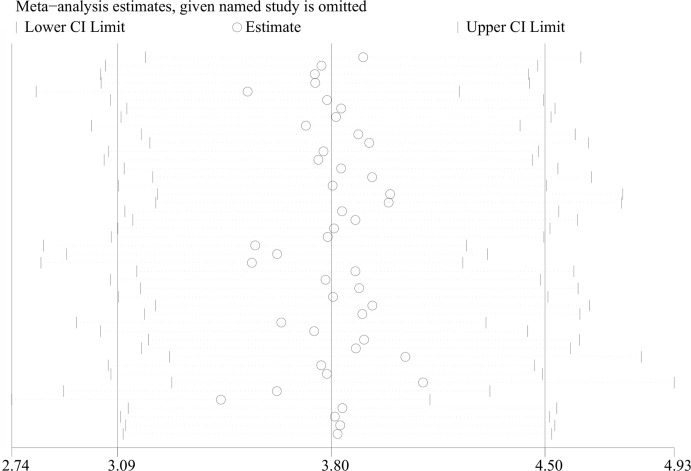
Sensitivity analysis of studies on leptin levels in patients with OSAS *versus* controls. OSAS, obstructive sleep apnea syndrome.

All 34 articles reported leptin levels in in 1485 OSA patients and 1201 controls. I^2^, an index of study heterogeneity, was 97.3%; thus, we selected a random-effects model to synthesize the data. The outcome of the meta-analysis showed that patients with OSA had significantly higher leptin levels compared to controls (WMD=3.80 ng/ml, 95%CI=3.09-4.50, *P*<0.00001; **Figure 2**). Furthermore, we conducted a series of sensitivity analyses to explore the stability of the pooled data. No significant differences were found after sequentially omitting studies, suggesting the reliability of this meta-analysis result (**Figure 3**). Different sample types may lead to increased heterogeneity; therefore, we analyzed serum leptin and plasma leptin levels separately (**Table 3**).

**Table 3:** Subgroup analyses of leptin levels in OSAS and controls.

Subgroup analysis of plasma levels (n)	WMD ng/ml (95% CI), P-value, I^2^ (%), Ph	Subgroup analysis of serum levels (n)	WMD ng/ml (95% CI), P-value, I^2^ (%), Ph
Overall (13)		Overall (32)	
Ethnicity		Ethnicity	
Caucasian (13)	3.78 (2.31,5.25), <0.0001, 98.20%, <0.0001	Caucasian (20)	5.58 (4.13,7.02), <0.0001, 94.50%, <0.0001
Asian (0)		Asian (11)	2.64 (1.10,4.18), 0.001, 91.6%, <0.0001
Latino (0)		Latino (1)	NA
Assay approaches		Assay approaches	
ELISA (6)	4.45 (2.75,6.16), <0.0001, 98.50%, <0.0001	ELISA (25)	3.46 (2.60,4.32), <0.0001, 93.80%, <0.0001
RIA (5)	2.68 (-0.78,6.13), 0.129, 93.30%, <0.0001	RIA (7)	6.30 (3.07,9.52), <0.0001, 86.40%, <0.0001
Mass spectrometry (2)	8.54 (-15.23,32.32), 0.481, 92.50%, <0.0001		
BMI		BMI	
Obese (5)	2.87 (-1.63,7.37), 0.211, 78.70%, 0.001	Obese (13)	3.23 (1.78,4.69), <0.0001, 77.80%, <0.0001
Non-obese (4)	6.12 (-1.08,13.33), 0.096, 97.50%, <0.0001	Non-obese (6)	4.35 (1.43,7.26), 0.003, 94.00%, <0.0001
Mixed (4)	2.32 (0.83,3.81), 0.002, 98.60%, <0.0001	Mixed (13)	4.31 (2.93,5.69), <0.0001, 95.80%, <0.0001
Degree of severity		Degree of severity	
Mild-to-moderate (4)	0.92 (-0.42,2.26), 0.178, 98.20%, <0.0001	Mild-to-moderate (9)	2.21(1.09,3.32), <0.0001, 90.40%, <0.0001
Severity (7)	6.74 (2.05,11.43), 0.005, 95.10%, <0.0001	Severity (7)	4.43 (1.98,6.88), <0.0001, 95.00%, <0.0001
Any severity (2)	7.44 (-9.05,23.93), 0.376, 97.40%, <0.0001	Any severity (16)	4.74 (2.68,6.79), <0.0001, 93.90%, <0.0001
Age		Age	
Adult (9)	5.13 (1.93,8.33), 0.002, 98.30%, <0.0001	Adult (27)	3.68 (2.83,4.54), <0.0001, 93.50%, 0.0001
Nonage (4)	2.05 (-1.43,5.52), 0.248, 98.20%, <0.0001	Nonage (5)	4.50 (0.19,8.82), 0.041, 93.80%, <0.0001
Study design		Study design	
CCS (11)	5.03 (2.04,8.01), 0.001, 97.90%, <0.0001	CCS (20)	4.74(3.67,5.81),<0.0001, 95.9%, <0.0001
CS (0)	NA	CS (1)	NA
CSS (2)	1.87 (-2.16,5.89), 0.364, 99.30%, <0.0001	CSS (11)	2.23(0.81,3.64), 0.002, 61.2%, 0.004
PSG type		PSG type	
Portable device (2)	0.13(-3.24,3.49), 0.941, 32.40%, 0.224	Portable device (3)	3.12 (0.64,5.59),0.013, 83.80%, 0.002
Lab PSG (11)	4.32 (2.73,5.91), <0.0001, 98.50%, <0.0001	Lab PSG (29)	4.02 (3.10,4.94),<0.0001, 94.20%, <0.0001

95%CI, 95% confidence interval; BMI, body mass index; ELISA, enzyme-linked immunosorbent assay; NA, not available; OSAS, obstructive sleep apnea syndrome; RIA, radioimmunoassay; WMD, weighted mean difference.

#### 3.3.2 Serum Leptin Levels in OSA Patients and Controls

In our meta-analysis, 32 eligible observational studies investigated the relationship between serum leptin concentration in patients with OSA and those without. A relationship between the serum leptin level and the presence of OSA was confirmed (WMD=3.90 ng/ml, 95%CI=3.04-4.75, *P<*0.0001; **Figure 2**), and we chose the random-effects model owing to the high level of heterogeneity (I^2^ = 93.8%; **Table 3**).

#### 3.3.3 Plasma Leptin Levels in OSA Patients and Controls

**Figure 2** shows the results of 13 observational studies describing plasma leptin levels. The pooled analysis comparing cases and controls illustrated that plasma leptin levels in individuals with OSA were remarkably higher than those in controls (WMD=3.78 ng/ml, 95%CI=2.31-5.25, *P*<0.0001; **Figure 2**).

### 3.4 Subgroup Analyses of Plasma Leptin Levels

#### 3.4.1 Age

Four studies analyzed plasma leptin concentrations in children and adolescents with and without OSAS. No significant difference was found in young patients with OSAS (WMD=2.05 ng/ml, 95%CI=−1.42-5.52, *P*=0.248) compared to controls. Nine studies compared plasma leptin concentrations between adults with and without OSAS and identified substantially higher leptin levels in adults with OSAS (WMD=5.13 ng/ml, 95%CI=1.93-8.33, *P*=0.002; **Table 3**).

#### 3.4.2 Body Mass Index

All included studies reported the BMI values for each patient. Therefore, we performed a meta-analysis of the articles according to obese, non-obese, and mixed groups. Some studies did not strictly distinguish between obese and non-obese populations, so we classified these studies as mixed groups. Four studies reported the plasma leptin levels in non-obese patients with OSA, and the plasma leptin concentrations did not differ between case and healthy control groups (WMD=6.12 ng/ml, 95%CI=−1.08-13.33, *P*=0.096; **Table 3**). Similar results were obtained in five studies providing the plasma leptin levels in obese individuals with OSA (WMD=2.87 ng/ml, 95%CI=−1.63-7.37, *P*=0.211). However, the remaining four studies with mixed groups showed remarkable differences between patients with OSA and healthy controls (WMD=2.32 ng/ml, 95%CI=0.83-3.81, *P*=0.002; **Table 3**).

#### 3.4.3 OSA Severity

A subgroup meta-analysis based on OSA severity was also performed. Four studies examined the association between plasma leptin levels and mild-to-moderate OSA, and seven studies included patients with severe OSA. Two studies provided data which cannot be classified according to degrees of severity. We defined the last two studies as any severity group. The results of our analysis revealed that the plasma leptin levels in patients with severe OSA were remarkably higher than those in controls (WMD=6.74 ng/ml, 95%CI=2.05-11.43, *P*=0.005). The results of the other subgroup analyses are presented in **Table 3**.

#### 3.4.4 Assay Approach

Considering the different assay techniques to determine leptin concentrations, subgroup analyses were carried out according to the assay type. Six studies assessed plasma leptin levels using enzyme-linked immunosorbent assays (ELISAs), and their results suggested that the plasma leptin concentration was higher in individuals with OSA compared to those in controls (WMD=4.45 ng/ml, 95%CI=2.75-6.16, *P*<0.0001; **Table 2**). Five studies determined the plasma leptin using radioimmunoassays (RIAs), and their results indicated that plasma leptin levels did not differ between individuals with OSA and controls (WMD=2.68 ng/ml, 95%CI=−0.78-6.13, *P*=0.129; **Table 3**). Two studies used high-performance liquid chromatography-tandem mass spectroscopy to detect leptin, and the leptin levels were only significantly different in obese patients with OSA (14) (**Table 3**).

#### 3.4.5 Ethnicity

The subgroup analysis of plasma leptin levels in patients with OSAS is summarized in **Table 2**. The main subgroup population comprised Caucasians. The pooled analysis demonstrated that higher plasma leptin concentrations were observed in Caucasian patients with OSA than in corresponding controls (WMD=3.78 ng/ml, 95%CI=2.31-5.25, *P*<0.0001). Only serum leptin levels were determined in other ethnicities; thus, no comparative data exist for plasma leptin levels in other ethnicities (**Table 3**).

#### 3.4.6 Study Design

Considering that variations in the design of included studies would affect the heterogeneity of the effect, we carried out a corresponding subgroup analysis. Compared to controls, for participant with OSA, the plasma leptin levels were significantly higher in case-control studies (WMD=5.03 ng/ml, 95%CI=2.04-8.01, *P*<0.0001). However, no difference was documented between OSA patients and controls in cross-sectional studies (WMD=1.87 ng/ml, 95%CI=-2.16-5.89, *P*=0.364) (**Table 3**).

#### 3.4.7 PSG Type

We conducted a subgroup analysis based on difficult PSG types used for diagnosing OSA. Eleven studies provided data on the plasma leptin levels in OSA patients diagnosed by lab PSG, and the results reveal that the plasma leptin levels were higher in these OSA patients than in the controls (WMD=4.32 ng/ml, 95%CI=2.73,5.91, *P*<0.0001). Two studies provided data on the plasma leptin levels in OSA patients diagnosed by portable device, and the results reveal that there was no difference in levels of leptin between OSA patients and controls (WMD=0.13 ng/ml, 95%CI=-3.24-3.49, *P*=0.941) (**Table 3**).

### 3.5 Subgroup Analysis of Serum Leptin Levels

#### 3.5.1 Age

Five studies compared serum leptin concentrations between children and adolescents with and without OSAS and found significantly higher leptin levels in those with OSAS (WMD=4.50 ng/ml, 95%CI=0.19-8.82, *P*=0.041). A total of 27 studies compared serum leptin concentrations between adults with and without OSAS and found significantly higher leptin levels in those with OSAS (WMD=3.68 ng/ml, 95%CI=2.83-4.54, *P*<0.0001; **Table 3**).

#### 3.5.2 Body Mass Index

We performed a meta-analysis of 32 studies according to the BMI. Six articles provided data on serum leptin levels in non-obese individuals with OSA, and the results of our analysis showed that the serum leptin concentration was significantly higher in non-obese individuals with OSA than in controls (WMD=4.35 ng/ml, 95%CI=1.43-7.26, *P*=0.003). Data on serum leptin levels in obese patients with OSA were available in 13 studies, and there were remarkable differences between obese individuals with OSA and controls. Another 13 studies did not strictly distinguish between obese and non-obese populations, so we classified these studies as mixed groups. The results demonstrated that the serum leptin level was remarkably higher in the mixed group than in the control group (**Table 3**).

#### 3.5.3 OSA Severity

Numerous studies have reported that serum leptin levels strongly correlate with OSA severity. In this meta-analysis, nine and seven articles reported serum leptin concentrations in patients with mild-to-moderate and severe OSA, respectively. However, there were 16 studies providing data which cannot be classified according to degrees of severity. We defined these studies as any severity group. The results of our analysis showed that the serum leptin concentrations in patients with mild-to-moderate OSA were remarkably higher than those in controls (WMD=2.21 ng/ml, 95%CI=1.09-3.32, *P*<0.0001). Eight studies supplied data on serum leptin levels according to OSA severity, showing that the serum leptin concentrations in patients with OSA were substantially higher than those in controls (WMD=4.43 ng/ml, 95%CI=1.98-6.88, *P*<0.0001). We also found that the serum leptin level in any severity group was remarkably higher than that in the control group (**Table 3**).

#### 3.5.4 Assay Approach

In 25 articles, the serum leptin levels were measured using ELISAs, and the results of our analysis suggested that in these studies, the serum leptin levels were higher in individuals with OSA than in controls (**Table 3**). Seven studies reported that serum leptin levels were assessed using RIAs. In these studies, serum leptin levels were higher in individuals with OSA than in controls (WMD=6.30 ng/ml, 95%CI=3.07-9.52, *P*<0.0001; **Table 3**).

#### 3.5.5 Ethnicity

The subgroup analysis of the pooled WMD for serum leptin levels in Caucasian, Asian, and Latino participants with OSAS indicated significant differences compared to the respective controls. Serum leptin levels in OSAS patients of these three ethnicities were higher than those in the respective control groups (**Table 3**).

#### 3.5.6 Study Design

Compared to controls, for participant with OSA, the serum leptin levels were significantly higher in case-control studies (WMD=4.74 ng/ml, 95%CI=3.67-5.81, *P*<0.0001). Similarly, levels of leptin in the OSA group were higher compared to those of the control group in cross-sectional studies (WMD=2.23 ng/ml, 95%CI=0.81-3.64, *P*=0.002) (**Table 3**).

#### 3.5.7 PSG Type

Twenty-nine studies provided data on the serum leptin levels in OSA patients diagnosed by lab PSG, and the results reveal that the serum leptin levels were higher in these OSA patients than in the controls (WMD=4.02 ng/ml, 95%CI=3.10-4.94, *P*<0.0001). Three studies provided data on the serum leptin levels in OSA patients diagnosed by portable device, and the results reveal that that the serum leptin levels were higher in these OSA patients than in the controls (WMD=3.12 ng/ml, 95%CI=0.64-5.59, *P*=0.013) (**Table 3**).

### 3.6 Meta-Analysis of the Relationship of Plasma/Serum Leptin Concentration With the AHI

**Figure 4:**
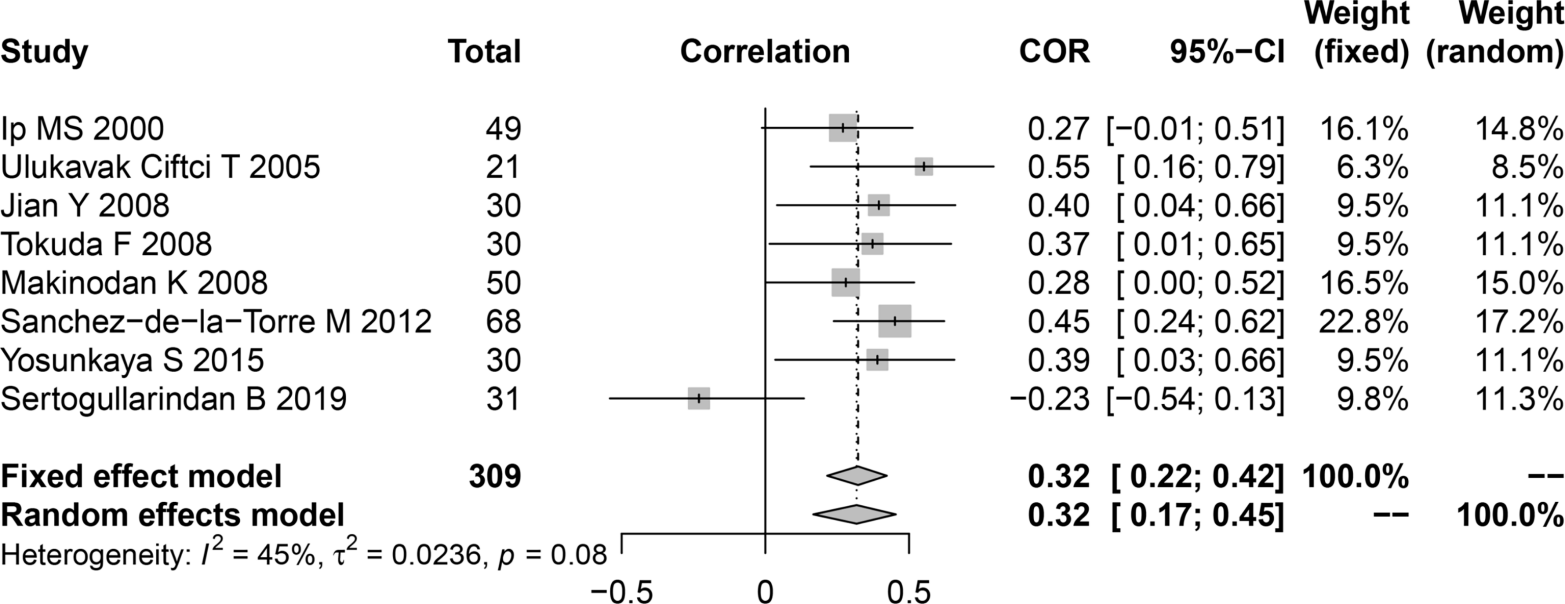
Funnel plot of effect sizes measured as correlations between serum/plasma leptin levels and AHI. AHI, apnea-hypopnea index.

Among the included publications, eight studies examined the relationship between AHI and serum leptin in patients with OSA and reported the Pearson’s or Spearman’s correlation coefficients. The AHI was often chosen as the measure to evaluate OSAS, as this parameter captures the OSAS severity. We performed a pooled analysis of the relationship between leptin levels and AHI among individuals with OSA using the R package meta. The effect size was 0.32 (95%CI=0.22-0.42, *P*<0.001), and heterogeneity was not significant (I^2^ = 45%, *P*=0.08; **Figure 4**). The meta-analysis of the correlation coefficients revealed a positive association between plasma/serum leptin levels and AHI.

### 3.7 Meta-Regression

The I^2^ value of all included studies was 97.3%, suggesting high study heterogeneity. Thus, we applied meta-regression analyses to identify potential sources of this heterogeneity.

The meta-regression data of serum leptin concentrations are presented in **Table 4**. Age, BMI, disease severity, assay approach, study design, PSG type, and ethnicity had no remarkable independent impact on serum/plasma leptin levels, as shown in **Table 4**. Other unknown factors influencing heterogeneity may have led to differences in the relationship between serum leptin concentration and OSA.

**Table 4:** Meta-regression analysis of variables predicting serum and plasma levels of leptin.

Factors	Samples	R	Adjusted R^2^	*P*
Age	serum	-0.064	-0.004	0.981
	plasma	1.001	-0.009	0.859
BMI	serum	-0.618	-0.003	0.375
	plasma	-0.191	-0.011	0.926
Severity	serum	-0.150	0.004	0.841
	plasma	0.293	0.011	0.905
Ethnicity	serum	-2.565	0.009	0.113
	plasma	—	—	—
Assay approaches	serum	-2.820	-0.001	0.220
	plasma	2.475	-0.008	0.512
Study design	serum	-2.143	0.049	0.201
	plasma	-3.562	-0.086	0.605
PSG type	serum	-1.351	-0.043	0.655
	plasma	-4.452	-0.063	0.529

BMI, body mass index.

### 3.8 Trial Sequential Analysis

**Figure 5:**
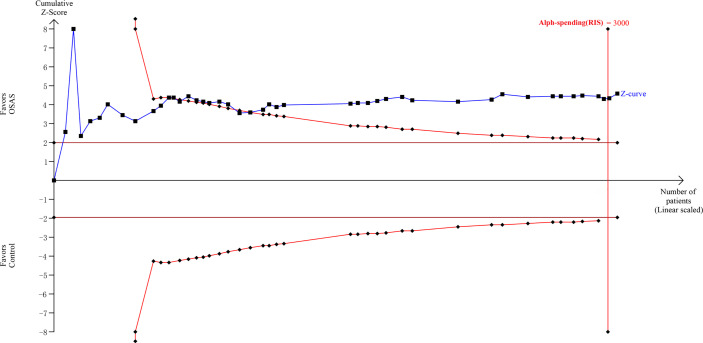
Trial sequential analysis.

The TSA showed that the cumulative Z curves crossed both the conventional boundary and the trial sequential monitoring boundary, and they surpassed the RIS as well (**Figure 5**). Therefore, the results are reliable, and further trials are not required.

### 3.9 Publication Bias

**Figure 6:**
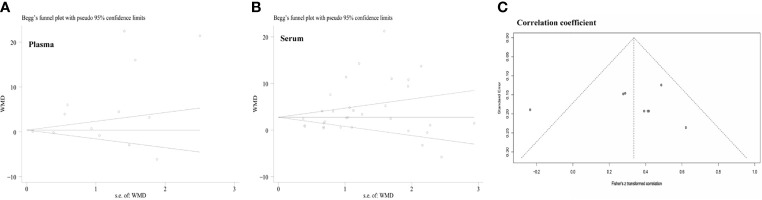
Funnel plots were employed to assess the publication bias among the included studies examining the relationship of leptin levels with OSAS. **(A)** Funnel plot of the plasma leptin levels in patients with OSAS *versus* the control group. **(B)** Funnel plot of the serum leptin levels in patients with OSAS *versus* the control group. **(C)** Funnel plot of the association between the correlation coefficient and AHI. AHI, apnea-hypopnea index; OSAS, obstructive sleep apnea syndrome.

A funnel plot was generated to assess the possibility of publication bias among the enrolled studies involving the relationship between leptin concentration and OSA. The Begg’s and Egger’s tests did not identify any publication bias across the studies of patients with OSA (plasma levels: Egger’s test [*P*=0.577] and Begg’s test [*P*=0.502], **Figure 6A**; serum levels: Egger’s test [*P*=0.654] and Begg’s test [*P*=0.224], **Figure 6B**). The Egger’s test of the funnel plot evaluating the association between the correlation coefficient and AHI also detected no evidence of publication bias (*P*=0.078; **Figure 6C**).

## 4 Discussion

The present meta-analysis of 45 studies evaluated the serum (27 studies of adults and 5 of children) and plasma (9 studies of adults and 4 of children and adolescents) leptin levels and compared these values between individuals with OSAS and controls. The analysis showed that both plasma and serum leptin levels in adults with OSAS were significantly higher than the corresponding levels in the control group. Similarly, serum leptin levels were significantly higher in children with OSAS than in controls. However, there was no significant difference in plasma leptin levels between children and adolescents with and without OSA. This may be related to the different samples included in the meta-analysis of plasma leptin concentrations, which included samples of adolescents leading to different meta-analysis results for serum and plasma leptin levels. Furthermore, subgroup analyses were conducted according to the study design and PSG type, and the serum leptin levels were higher in the OSA patients. Moreover, the meta-analysis of the correlation coefficients revealed a pattern of positive associations between plasma/serum leptin levels and AHI. These results were further supported by TSA, demonstrating that the current sample size was adequate. However, further well-designed large-scale studies involving various ethnicities and geographical contexts are encouraged. The present conclusions have clinical importance because an elevated leptin level could be a risk factor for the development of cardiovascular disease in adults with OSAS (47). It is crucial to understand the mechanism which drive these changes in leptin levels because leptin has been reported to be related with an increased risk of myocardial infarction, hemorrhagic stroke, abnormal fibrinolysis in men and postmenopausal women (48–50) . These disorders are also typical complications among OSA patients. In addition, these results have practical importance because, in addition to routine checks on sleep and sleep-disordered breathing, both children and adults with OSAS need a thorough monitoring of metabolic level. Such detailed analyses could enable better understanding of leptin changes in OSA patients, building confidence in OSA risk-assessment.

Previously it was shown that OSAS can alter the levels of several hormones (12). Obesity and OSAS can lead to elevated levels of the adipose-derived hormone leptin, which increases metabolism (40). Leptin resistance is a major pathophysiological factor of obesity (51). Obese individuals do not lack leptin; rather, they display higher circulating levels of leptin, and these elevated levels are related to leptin resistance and impaired leptin signaling in the brain (52). OSAS and obesity are bidirectionally linked. Many studies have reported that patients with OSAS have higher leptin levels than control subjects. Although the exact mechanism of OSAS-induced impact on leptin levels is not clear, both sleep deprivation and hypoxemia are thought to be critical causative factors (53). Our combined results also indicate that plasma/serum leptin levels are higher in patients with OSA than in the control group. In the subgroup analysis according to the BMI, the plasma leptin concentration for OSA patients with a BMI of <30 kg/m^2^ was not different compared to controls, whereas the serum leptin level in OSA patients with a BMI of <30 kg/m^2^ was higher than that in healthy controls, indicating that OSA might influence the leptin level. Similarly, serum leptin levels in obese patients with OSA were higher than those in obese patients without OSA. The reason for the observed difference between plasma and serum leptin levels may be that too few studies (only four) were included in the BMI<30 group for the meta-analysis of plasma leptin levels. In general, our study results indicated that both plasma and serum leptin levels were increased in OSAS regardless of disease severity and assay approach, suggesting that leptin might be a risk factor for OSAS. Interestingly, the increase in plasma/serum leptin level showed subtle alterations in different ethnicities. The ethnicity may influence plasma/serum leptin levels in patients with OSAS. Different human polymorphisms of leptin gene and leptin receptor gene have been characterized (54–56). The synthesis of this hormone is regulated by genetic polymorphisms in the leptin gene (57). Sahin et al. (58) and Mammès et al. (59) suggested that the LEP rs2167270 A allele is associated with increased serum leptin levels in Caucasians. Similarly, Ren et al. (60) found that LEP rs8179183 is associated with serum leptin levels and overweight/obesity in Chinese adolescents.

Leptin, a 16-kD adipokine, regulates weight centrally and is also involved in a cytokine network that governs the inflammatory immune response by releasing proinflammatory cytokines (61). The underlying mechanism of the relationship between plasma/serum leptin concentration and OSA remains unclear. Based on the source, metabolism, and other factors influencing leptin, we summarize here several seemingly reasonable biological explanations. First, researchers surmise that, similar to hyperleptinemia in patients with obesity and metabolic syndrome, leptin resistance is present in patients with OSAS (20). As leptin resistance prevents leptin from performing its normal physiological function, fat distribution is imbalanced, and excess fat is deposited in the upper airway and viscera, contributing to the collapse of the airway during sleep and the onset of OSAS, while leptin concentrations increase as a compensatory mechanism. One study demonstrated that OSAS increases leptin levels independently of obesity (62). Likewise, Ip et al. (18) studied serum leptin levels in obese patients with OSAS after nasal continuous positive airway pressure ventilation (nCPAP). They found distinct hyperleptinemia in OSAS patients before treatment, but plasma leptin levels returned to normal values following nCPAP treatment while the patients maintained their body weights. The results of the present study also showed a moderate positive correlation between leptin concentration and AHI, which supports the above hypothesis. Second, hypoxia is the most important pathophysiological change in patients with OSAS (63). As AHI increases, oxygen saturation decreases significantly at night and hypoxemia occurs, which causes activation of the sympathetic nervous system, elevated angiotensin levels, and blood pressure fluctuations, all of which can lead to increased plasma leptin levels (48, 64). In patients with OSA, repetitive apnea episodes aggravate the hypoxemia and CO_2_ retention, both of which augment sympathetic activity and reduce parasympathetic activity (48, 64). It is tempting to speculate that sympathetic nerve activity may affect the secretion of leptin. Third, the effect of leptin on respiration is not limited to the regulation of lipid metabolism; it has a more direct effect on the respiratory system. The leptin replacement test in leptin-deficient *ob*/*ob* mice showed that leptin is not only a growth factor during lung development but also a neuroregulatory factor in the respiratory center (65). Studies in animal models have shown that leptin is a powerful stimulant of ventilation that can prevent respiratory depression in obese animals. There is accumulating evidence that leptin affects the contraction of the upper airway during sleep (66), suggesting that elevated leptin levels in the central nervous system may prevent the occurrence of OSAS. The increase in serum leptin levels in patients with OSAS may be a compensatory protective mechanism to prevent respiratory depression (67). Therefore, hyperleptinemia and leptin resistance may play important roles in the occurrence and development of OSAS. In addition to obesity, lipid metabolism disorders, systemic inflammatory responses, and oxidative stress, leptin levels were also affected by sex differences, circadian rhythm of leptin secretion, metabolic disease, and lifestyle, among others (68–70). However, because these parameters cannot be extracted from all or at least most of the included studies, it is impossible to carry out further subgroup analyses. There are also diverging views on the effects of leptin on OSAS. Pamuk et al. (71) reported that serum leptin levels of non-obese individuals and non-obese patients with mild OSA are not significantly different and suggested that obesity is an important factor affecting serum leptin levels. A study on the role of leptin in children with OSA found no significant difference in leptin levels between OSA and non-OSA groups, and after correction for obesity factors, the correlation between leptin and oxygen desaturation index was eliminated (43). These results differ from the results of this meta-analysis, maybe because a single-study sample is relatively smaller than that of the present meta-analysis or maybe due to differences in the included populations. The relationship between leptin and OSAS remains controversial, and the underlying physiological mechanism of the effect of leptin on OSA warrants further studies.

The overall heterogeneity was high in this meta-analysis (I^2^ = 97.3%); nevertheless, the sensitivity assessment indicated that no single study significantly influenced the pooled WMD, suggesting that our data are robust. Meta-regression analyses demonstrated that age, BMI, disease severity, assay approach, study design, PSG type, and ethnicity had no remarkable impact on heterogeneity in this meta-analysis. The heterogeneity of each subgroup remained high after subgroup analyses for these factors. This suggests that several unknown factors contributed to the observed study heterogeneity, such as different experimental reagents, physiques of the patients, or their dietary habits.

This meta-analysis has some strengths in examining the plasma/serum leptin levels in patients with OSAS. First, our results demonstrate that the plasma/serum leptin levels may be a clinically beneficial biological marker, which may aid clinicians in comprehensively diagnosing OSAS and assessing the severity of OSAS. For the first time, we combined Pearson’s correlation coefficients and analyzed the correlation between AHI and leptin levels, thereby demonstrating the relationship between leptin levels and AHI from the perspective of evidence-based medicine. Changes in plasma/serum leptin concentrations may facilitate our understanding of the metabolic mechanisms involved in OSAS. Hirotsu et al. (72) reported a possible participation of leptin in pathophysiological and inflammatory processes in OSA progression. Leptin may be a critical link associated with coexisting metabolic disorders. Manzella et al. (73) also demonstrated that leptin concentrations are positively associated with AHI values, proposing leptin as a biosignature of disease severity. The results of our study are consistent with those of previous reports. Second, this is the most comprehensive meta-analysis of relevant literature to provide robust findings. Although a few reports have previously established a link between leptin and OSA, they only studied leptin receptor gene polymorphisms or effects of continuous positive airway pressure on leptin levels in patients with OSA (74–80). We included cross sectional, case-control cohort studies to analyze the different leptin levels in OSA patients and controls. Our study had a larger sample size (2686 participants), and included more novel researches. Third, all included articles were of medium or high quality, making the assessment more reliable. Fourth, we excluded the potential impact of differences in specimen sources by analyzing serum and plasma leptin levels separately, ruling out this confounding factor.

Despite the novelty of our findings, the following limitations should be considered. First, none of the studies were adjusted to reflect possible confounding factors such as stable daily routine, smoking, or alcohol consumption. And there are only two articles in the original literatures (26, 44) were concerning on male patients with OSA. Although we tried our best to control the confound founding factors, some potential confounding factors may affect the conclusion more or less. Second, since this study lacked effective longitudinal cohort studies, we could not infer causality of the association between plasma/serum leptin levels and OSA. Third, the majority of the included studies had a sample size of fewer than 100 cases, which had insufficient power to analyze the association between leptin levels and OSAS.

## 5 Conclusion

It is concluded that a high plasma leptin level may be correlated with OSA, and the serum/plasma leptin level positively correlates with AHI. Furthermore, ethnicity has an impact on the association between OSAS and leptin levels. Finally, more elaborate studies are required in the future to ascertain the association between leptin levels and OSAS risk.

## Data Availability Statement

The original contributions presented in the study are included in the article/supplementary material. Further inquiries can be directed to the corresponding author.

## Author Contributions

Data curation: JH. Formal analysis: XL. Methodology: XL. Project administration: XL. Resources: JH. Supervision: XL. Validation: JH. Writing-original draft: XL. All authors contributed to the article and approved the submitted version.

## Conflict of Interest

The authors declare that the research was conducted in the absence of any commercial or financial relationships that could be construed as a potential conflict of interest.

## Publisher’s Note

All claims expressed in this article are solely those of the authors and do not necessarily represent those of their affiliated organizations, or those of the publisher, the editors and the reviewers. Any product that may be evaluated in this article, or claim that may be made by its manufacturer, is not guaranteed or endorsed by the publisher.
